# Body weight changes in patients with type 2 diabetes and a recent acute coronary syndrome: an analysis from the EXAMINE trial

**DOI:** 10.1186/s12933-021-01382-8

**Published:** 2021-09-14

**Authors:** João Pedro Ferreira, Patrick Rossignol, George Bakris, Cyrus Mehta, William B. White, Faiez Zannad

**Affiliations:** 1 Centre DInvestigation Clinique-Plurithématique Inserm CIC-P 1433, Inserm U1116, CHRU Nancy hopitaux de Brabois, F-CRIN INI-CRCT (Cardiovascular and Renal Clinical Trialists), Université de Lorraine, Institut Lorrain du Coeur et des Vaisseaux Louis Mathieu, 4 Rue du Morvan, 54500 Vandoeuvre lès Nancy, France; 2 Cardiovascular Research and Development Center, Faculty of Medicine, University of Porto, Porto, Portugal; 3 Department of Medicine, American Heart Association Comprehensive Hypertension Center, University of Chicago, Chicago, IL USA; 4 Cytel Corporation, Cambridge, MA USA; 5 Calhoun Cardiology Center, University of Connecticut School of Medicine, Farmington, CT USA

**Keywords:** Type 2 diabetes, Weight changes, Cardiovascular outcomes

## Abstract

**Background:**

Patients with type 2 diabetes (T2D) may experience frequent body weight changes over time. The prognostic impact of these weight changes (gains or losses) requires further study.

**Aims:**

To study the associations between changes in body weight (intentional or unintentional) with subsequent outcomes.

**Methods:**

The EXAMINE trial included 5380 patients with T2D and a recent acute coronary syndrome, who were randomized to alogliptin or placebo. Time-updated Cox models and mixed effects models were used to test the associations between changes in body weight and subsequent outcomes over a median follow-up of 1.6 (1.0–2.1) years.

**Results:**

During the post-randomization follow-up period, 1044 patients (19.4%) experienced a weight loss ≥ 5% of baseline weight, 2677 (49.8%) had a stable weight, and 1659 (30.8%) had a ≥ 5 % weight gain. Patients with weight loss were more frequently women and had more co-morbid conditions. In contrast, patients who gained ≥ 5% weight were more frequently men with less co-morbid conditions. A weight loss ≥ 5% was independently associated with a higher risk of subsequent adverse outcomes, including all-cause mortality: adjusted HR (95% CI) = 1.79 (1.33–2.42), P < 0.001. Similar associations were found for cardiovascular mortality, the composite of cardiovascular mortality or heart failure hospitalization, and the primary outcome. A weight gain ≥ 5% was independently associated with an increase in the risk of subsequent cardiovascular mortality or heart failure hospitalization only: adjusted HR (95% CI) = 1.34 (1.02–1.76), P = 0.033.

**Conclusions:**

In patients with T2D who had a recent ACS/MI, a ≥ 5% loss of body weight was associated with a higher risk of subsequent cardiovascular events and mortality.

**Supplementary Information:**

The online version contains supplementary material available at 10.1186/s12933-021-01382-8.

## Introduction

Low body weight or low body mass index (BMI) has been associated with poor outcomes in patients with type 2 diabetes [1, 2]. Although, excess weight and obesity increase the risk of T2D and cardiovascular diseases [3–5], among patients who have developed T2D and other chronic conditions, obesity is not associated with poor outcomes [2, 6]. This phenomenon has been referred to as the “obesity paradox” and may indicate that patients with lower versus higher body weight may experience more cachexia, wasting and frailty [7]. Furthermore, patients with T2D may experience “dynamic” weight changes over the course of the disease, which may have prognostic impact (particularly weight loss), independently of the BMI [2].

An important distinction is whether the weight loss was intentional or unintentional. Observational studies distinguishing intentional from unintentional weight loss suggest that weight loss may be a mortality risk factor regardless of intent to lose weight [8]. Large-scale trials in which an intentional weight loss program was part of the intervention improved diabetes control and cardiovascular risk factors but had a neutral effect on morbidity and mortality outcomes [9].

Using data from the Cardiovascular Outcomes Study of Alogliptin in Patients With Type 2 Diabetes and Acute Coronary Syndrome (EXAMINE) trial, that included patients with T2D who had a recent acute coronary syndrome (ACS) or myocardial infarction (MI) randomly assigned to either alogliptin or placebo [10], we explored the factors associated with changes in body weight (loss and/or gain), the effect of alogliptin on body weight over time, and he prognostic associations of weight changes.

## Methods

### Study design

Details of the EXAMINE trial design have been previously published [10, 11]. In short, a total of 5380 patients with T2D who had a recent ACS were randomly assigned to either alogliptin or placebo.

In the overall population, alogliptin significantly reduced glycated hemoglobin (HBA1c) without altering the rates of major adverse cardiovascular events over a median (percentile_25 − 75_) follow-up of 1.6 (percentile_25 − 75_: 1.0–2.1) years.

The steering committee, consisting of academic members and three nonvoting representatives of the sponsor (Takeda Development Center Americas), designed and oversaw the conduct of the trial. An independent data and safety monitoring committee monitored the trial and had access to the unblinded data.

### Study patients

Patients were eligible for enrolment if they had received a diagnosis of T2D, were receiving antidiabetic therapy (other than a DPP-4 inhibitor or GLP-1 analogue) and had an ACS (MI and unstable angina requiring hospitalization) within 15 to 90 days prior to randomization. Major exclusion criteria were a diagnosis of type 1 diabetes, unstable cardiac disorders (e.g., advanced heart failure, refractory angina, uncontrolled arrhythmias, critical valvular heart disease, or severe uncontrolled hypertension), active cancers, and dialysis within 14 days before screening.

Institutional Review Board approval was obtained, ethics approval was obtained at each participating site, and all patients provided informed consent to participate in the trial. The EXAMINE trial is registered with the clinicaltrials.gov number NCT00968708.

### Body weight changes

Body weight changes were calculated at each post-randomization study visit (1 month, 3 months, 6 months, 9 months, 12 months, and every 4 months thereafter) as the percentage (%) of weight change from randomization to study visit. Patients were further categorized by weight losses and gains during the follow-up (≥ 5% and ≥ 10%).

### Study outcomes

The primary endpoint in EXAMINE was a composite of death from cardiovascular causes, nonfatal MI, or nonfatal stroke, adjudicated by an independent committee blinded to treatment assignment. In the present analysis we also studied the association of weight changes with subsequent occurrence of cardiovascular death or HF hospitalization, cardiovascular death, and all-cause death.

### Statistical analysis

For descriptive statistics, EXAMINE patients were divided in those experiencing any weight loss or gain ≥ 5% and ≥ 10% vs. those not experiencing such weight changes. Patients’ characteristics are presented as mean ± standard deviation, median (percentile_25 − 75_), or numbers and percentages, as appropriate. The groups were compared using parametric or non-parametric tests for continuous variables and chi-square tests for categorical variables. To study the effect of alogliptin (vs. placebo) on weight over time, mixed effects linear regression models were used with continuous weight as outcome variable, and study visit, treatment (alogliptin or placebo), study visit by treatment interaction, diabetes duration, insulin, metformin, statins, current smoking, heart failure history, anemia, hypertension, body mass index (BMI), estimated glomerular filtration rate (eGFR), and diuretics as dependent variables, with random effects at the patient level, and unstructured covariance matrix to allow for correlation between measures. Mixed effects logistic regression models were performed to study the effect of alogliptin on weight loss or gain (≥ 5% and ≥ 10%) throughout the follow-up.

The impact of weight changes (≥ 5% and ≥ 10% loss or gain) on outcomes was studied with time-updated Cox models (that take into account the most recent value before an event), adjusting for treatment (alogliptin or placebo), diabetes duration, insulin, metformin, statins, current smoking, heart failure history, anemia, hypertension, BMI, eGFR, and diuretics. An interaction between weight changes and BMI was tested for each outcome, to assess whether the impact of weight changes on outcomes could vary by baseline BMI i.e., if it would be different in obese (BMI ≥ 30 Kg/m^2^) vs. non-obese (BMI < 30 Kg/m^2^) patients. The association between time-update BMI and subsequent outcomes was studied across quintiles of BMI (≤ 22, 23–25, 26–30, 31–35, > 35 Kg/m^2^) adjusting for the same variables above described (except BMI which is the variable of interest) and using the middle category (26–30 Kg/m^2^) as the referent category. The adjustment variables were selected for their prognostic importance, based on previous EXAMINE publications and differences found in Table 1 [12,13,14]. The last available value was carried forward whenever missing values occurred in the time-updated models. Two-tailed P values < 0.05 were considered statistically significant. Statistical analyses were performed using STATA® Statistical Software version 17.0 (STATA Corp, College Station, Texas).

## Results

### Baseline characteristics of the patients by weight changes

Of the 5380 randomized patients, 1044 (19.4%) experienced a weight loss ≥ 5% of baseline weight, 2677 (49.8%) had a stable weight within the 5% range, and 1659 (30.8%) gained weight ≥ 5% of their baseline weight. The histogram representing the weight changes throughout the follow-up is displayed in Additional file 1: Fig. S1. Compared to patients with a stable weight, those who lost ≥ 5% of their weight were more frequently women (38.0 vs. 31.5%), had higher body weight and BMI at baseline (84.5 vs. 83.6 Kg and 30.5 vs. 29.7 Kg/m^2^, respectively), had a more frequent history of peripheral artery disease (11.7 vs. 9.9%), atrial fibrillation (9.1 vs. 7.1%), anemia (30.0 vs. 23.3%), and kidney disease (eGFR < 60 ml/min: 33.8 vs. 28.4%), with a more frequent use of diuretics (41.4 vs. 36.9%) (Table 1). In contrast, patients who gained ≥ 5% weight were younger, more frequently men with lower weight and BMI at baseline, and less comorbid conditions including HF, peripheral artery disease, atrial fibrillation, anemia, and kidney disease (Table 1). Similar characteristics were observed for patients who had weight changes more than 10% of their baseline weight (Additional file 1: Table S1).

**Table.1:** Baseline characteristics of the patients by 5% weight change from baseline

Characteristic	Weight loss ≥ 5%	Stable weight	Weight gain ≥ 5%	P-value
N	1044	2677	1659	
Age, years	61.3 ± 10.0	61.7 ± 10.0	59.3 ± 9.6	< 0.001
Age > 65 year	391 (37.5%)	1052 (39.3%)	464 (28.0%)	< 0.001
Female, n (%)	397 (38.0%)	843 (31.5%)	489 (29.5%)	< 0.001
Diabetes duration, years	7.5 (2.8, 14.0)	7.1 (2.9, 13.3)	6.9 (2.4, 14.0)	0.43
Glycated hemoglobin, %	8.0 ± 1.1	8.0 ± 1.1	8.1 ± 1.1	0.030
Insulin, n (%)	300 (28.7%)	782 (29.2%)	523 (31.5%)	0.19
Metformin, n (%)	701 (67.1%)	1776 (66.3%)	1085 (65.4%)	0.63
Sulfonylureas, n (%)	445 (42.6%)	1270 (47.4%)	788 (47.5%)	0.019
Thiazolidinediones, n (%)	38 (3.6%)	49 (1.8%)	44 (2.7%)	0.004
Weight at baseline, Kg	84.5 ± 21.0	83.6 ± 18.6	78.5 ± 18.3	< 0.001
BMI at baseline, Kg/m^2^	30.5 ± 6.3	29.7 ± 5.4	28.4 ± 5.3	< 0.001
Race				< 0.001
White	735 (70.4%)	2051 (76.6%)	1123 (67.7%)	
Asian	232 (22.2%)	479 (17.9%)	378 (22.8%)	
Black	57 (5.5%)	79 (3.0%)	80 (4.8%)	
Other	20 (1.9%)	68 (2.5%)	78 (4.7%)	
Current smoker, n (%)	145 (13.9%)	374 (14.0%)	215 (13.0%)	0.62
Hypertension, n (%)	889 (85.2%)	2279 (85.1%)	1301 (78.4%)	< 0.001
HF history, n (%)	298 (28.5%)	836 (31.2%)	399 (24.1%)	< 0.001
Prior stroke, n (%)	85 (8.1%)	190 (7.1%)	113 (6.8%)	0.40
PAD, n (%)	122 (11.7%)	266 (9.9%)	126 (7.6%)	0.001
Atrial fibrillation, n (%)	95 (9.1%)	190 (7.1%)	91 (5.5%)	0.002
MI (index event), n (%)	794 (76.3%)	1993 (74.7%)	1365 (82.3%)	< 0.001
Unstable angina (index event), n (%)	246 (23.7%)	675 (25.3%)	293 (17.7%)	
Hemoglobin, g/dl	13.2 ± 1.6	13.6 ± 1.6	13.4 ± 1.5	< 0.001
Anemia, n (%)	312 (30.0%)	623 (23.3%)	433 (26.1%)	< 0.001
eGFR, ml/min/1.73m^2^	67.5 ± 21.2	71.1 ± 21.1	72.8 ± 21.8	< 0.001
eGFR < 60 ml/min, n (%)	353 (33.8%)	761 (28.4%)	451 (27.2%)	< 0.001
Heart rate, bpm	72.1 ± 11.2	70.8 ± 10.7	71.8 ± 10.7	< 0.001
SBP, mmHg	129.7 ± 17.4	129.9 ± 15.8	127.1 ± 17.3	< 0.001
SBP > 140/90 mmHg, n (%)	238 (22.8%)	545 (20.4%)	297 (17.9%)	0.007
Total cholesterol, mg/dl	146 (123, 148)	149 (126, 178)	143 (122, 173)	0.001
HDL cholesterol, mg/dl	42 (36, 48)	42 (36, 49)	42 (35, 48)	0.11
LDL cholesterol, mg/dl	72 (55, 97)	74 (55, 97)	70 (53, 94)	0.010
Triglycerides, mg/dl	138 (102, 192)	143 (104, 200)	139 (102, 191)	0.12
Antiplatelets at baseline, n (%)	1008 (96.6%)	2600 (97.1%)	1624 (97.9%)	0.10
Beta-blockers at baseline, n (%)	836 (80.1%)	2220 (82.9%)	1355 (81.7%)	0.12
Statins at baseline, n (%)	930 (89.1%)	2402 (89.7%)	1534 (92.5%)	0.003
CCBs at baseline, n (%)	244 (23.4%)	641 (23.9%)	312 (18.8%)	< 0.001
Diuretics at baseline, n (%)	432 (41.4%)	988 (36.9%)	594 (35.8%)	0.010
ACEi or ARB at baseline, n (%)	856 (82.0%)	2227 (83.2%)	1328 (80.0%)	0.033
Randomization to alogliptin	541 (51.8%)	1332 (49.8%)	828 (49.9%)	0.51

BMI, body mass index; PAD, peripheral artery disease; MI, myocardial infarction; eGFR, estimated glomerular filtration rate; SBP, systolic blood pressure; CCBs, calcium channel blockers; ACEi/ARBs, angiotensin converting enzyme inhibitors/angiotensin receptor blockers

### Effect of alogliptin on body weight

**Fig. 1:**
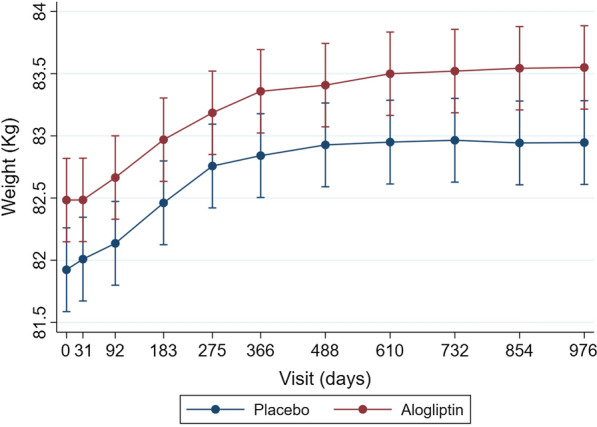
Effect of alogliptin (vs. placebo) on body weight throughout the follow-up

Patients randomized to alogliptin (vs. placebo) had a small increase in mean body weight throughout the follow-up of + 0.54 (95% CI 0.08 to 0.99) Kg, P = 0.020 (Fig. 1), but did not lead to increases in body weight of ≥ 5% or ≥ 10% (Additional file 1: Table S2).

### Body weight changes and subsequent outcomes

**Fig. 2:**
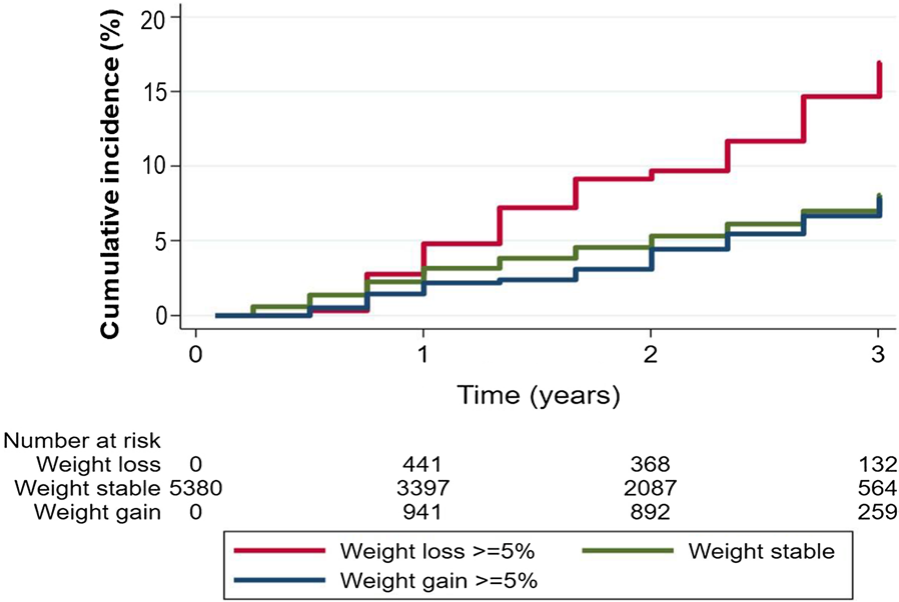
Association of a 5% weight change with subsequent mortality

Weight losses of ≥ 5% and ≥ 10% were independently associated with a higher risk of cardiovascular and all-cause mortality: weight loss ≥ 5% all-cause mortality adjusted HR (95% CI) = 1.79 (1.33–2.42), P < 0.001 and weight loss ≥ 10% all-cause mortality adjusted HR (95% CI) = 2.07 (1.31–3.25), P = 0.002 (Table 2 and Fig. 2). Similar associations were found for cardiovascular mortality, the composite of cardiovascular mortality or HF hospitalization, and the primary MACE outcome. A weight gain ≥ 5% was only independently associated with a small increase in the risk of subsequent cardiovascular mortality or HF hospitalization: adjusted HR (95% CI) = 1.34 (1.02–1.76), P = 0.033. A weight gain ≥ 10% was not associated with risk subsequent events. Baseline BMI did not influence the prognostic impact of weight changes (interaction P > 0.1 for all outcomes) (Table 2).

**Table.2:** Association of weight change with subsequent outcomes

Outcome/weight changes	Crude HR (95% CI)	Adj. HR (95% CI)*	P-value
All-cause death			
Weight loss ≥ 5 %	2.15 (1.61–2.87)	1.79 (1.33–2.42)	< 0.001
Stable weight	Ref.	Ref.	–
Weight gain ≥ 5 %	0.99 (0.73–1.32)	1.01 (0.74–1.36)	0.96
Weight loss ≥ 10 %	2.74 (1.78–4.25)	2.07 (1.31–3.25)	0.002
Stable weight	Ref.	Ref.	–
Weight gain ≥ 10 %	0.90 (0.54–1.52)	0.83 (0.50–1.41)	0.50
Cardiovascular death			
Weight loss ≥ 5 %	2.21 (1.57–3.10)	1.84 (1.30–2.61)	0.001
Stable weight	Ref.	Ref.	–
Weight gain ≥ 5 %	1.16 (0.83–1.62)	1.21 (0.86–1.70)	0.28
Weight loss ≥ 10 %	2.56 (1.50–4.37)	1.89 (1.10–3.26)	0.021
Stable weight	Ref.	Ref.	–
Weight gain ≥ 10 %	0.94 (0.51–1.73)	0.88 (0.48–1.63)	0.68
CVD/HFH			
Weight loss ≥ 5 %	2.61 (1.99–3.41)	2.19 (1.66–2.89)	< 0.001
Stable weight	Ref.	Ref.	–
Weight gain ≥ 5 %	1.29 (0.99–1.69)	1.34 (1.02–1.76)	0.033
Weight loss ≥ 10 %	3.59 (2.42–5.33)	2.83 (1.87–4.23)	< 0.001
Stable weight	Ref.	Ref.	–
Weight gain ≥ 10 %	1.07 (0.67–1.72)	1.00 (0.63–1.61)	0.99
Primary outcome			
Weight loss ≥ 5 %	1.87 (1.49–2.36)	1.67 (1.32–2.10)	< 0.001
Stable weight	Ref.	Ref.	–
Weight gain ≥ 5 %	0.91 (0.73–1.15)	0.94 (0.74–1.18)	0.57
Weight loss ≥ 10 %	2.53 (1.76–3.63)	2.14 (1.49–3.08)	< 0.001
Stable weight	Ref.	Ref.	–
Weight gain ≥ 10 %	0.84 (0.56–1.27)	0.82 (0.54–1.24)	0.34

CVD/HFH, composite of cardiovascular death or heart failure hospitalization; The primary outcome was a composite of myocardial infarction, stroke, or cardiovascular death

*Model adjusted on randomized treatment (alogliptin or placebo), age, sex, diabetes duration, insulin, metformin, statins, current smoking, heart failure history, anemia, hypertension, body mass index, estimated glomerular filtration rate, diuretics

The P-value is for the adjusted model

Baseline body mass index (BMI ≥ 30 vs. <30 Kg/m^2^) did not modify the associations between weight changes and subsequent outcomes:

P for interaction 5% weight change × BMI for all-cause mortality = 0.73

P for interaction 10% weight change × BMI for all-cause mortality = 0.27

P for interaction 5% weight change × BMI for cardiovascular mortality = 0.76

P for interaction 10% weight change × BMI for cardiovascular mortality = 0.70

P for interaction 5% weight change × BMI for cardiovascular mortality or heart failure hospitalization = 0.51

P for interaction 10% weight change × BMI for cardiovascular mortality or heart failure hospitalization = 0.17

P for interaction 5% weight change × BMI for the primary outcome = 0.58

P for interaction 10% weight change × BMI for the primary outcome = 0.70

### Body mass index and subsequent outcomes

Compared to patients with a BMI between 26 and 30 kg/m^2^, those with a BMI ≤ 22 kg/m^2^ had a higher risk of cardiovascular outcomes including all-cause and cardiovascular mortality: adjusted HR (95% CI) = 2.07 (1.29–3.30), P = 0.002 and = 1.78 (1.01–3.16), P = 0.047, respectively. Patients with a BMI > 30 kg/m^2^ (including those with a BMI > 35 kg/m^2^) had a lower risk of a primary outcome event: adjusted HR (95% CI) = 0.75 (0.60–0.96), P = 0.020 for patients with a BMI between 31 and 35 kg/m^2^ and = 0.61 (0.41–0.92), P = 0.017 for patients with a BMI > 35 kg/m^2^ (Additional file 1:Table S3).

## Discussion

The present study shows that patients who experienced a weight loss of 5% or greater had more co-morbid conditions (e.g., peripheral artery disease, atrial fibrillation, anemia, and chronic kidney disease) than those who did not lose or even gained weight. A weight loss of 5% or more of body weight was independently associated with a higher risk of cardiovascular events and mortality. Weight gain was only modestly associated with an increased risk of the cardiovascular composite that included mortality plus HF hospitalizations. This study also showed that a low BMI (≤ 22 kg/m^2^) was associated with a higher risk of subsequent events, whereas a high BMI (even > 35 kg/m^2^) was associated with a lower risk of primary outcome events. Alogliptin (vs. placebo) led to a slight (0.5 kg on average) increase in body weight throughout the follow-up and did not increase the odds of experiencing weight gains in excess of 5% of baseline weight.

Weight loss has been associated with an increased mortality risk in patients with T2D. Data from the ORIGIN trial, including patients with established cardiovascular risk factors and T2D or pre-diabetes, also showed that weight loss was associated with increases in the risk of cardiovascular events and mortality, whereas weight gain was not associated with adverse outcomes [2]. Moreover, the ORIGIN trial also showed that patients with a BMI < 22 kg/m^2^had an increased risk of adverse outcomes and that overweight or obese patients had a lower risk of cardiovascular events [2]. Long-term follow-up data from 444 patients in the Diabetes Care in General Practice study, suggested that weight loss was associated with an increased risk of mortality, regardless of the intention to lose or not to lose weight [8]. Similar associations were found in patients with a recent MI complicated with HF [6], and in patients with HF and a reduced ejection fraction regardless of the T2D status [15, 16]. Our study confirms these findings in a unique high cardiovascular risk T2D population with recent acute coronary syndromes and regardless of the baseline BMI (i.e., a weight loss ≥ 5% may be associated with a poor prognosis even in obese patients at baseline (with a BMI > 30 kg/m^2^). However, data from large randomized controlled trials do not confirm these observational findings (see below), suggesting that in observational studies it may be difficult to distinguish intentional from unintentional weight loss and control for confounding i.e., the findings from observational studies likely represent a “reverse causation” or “reverse epidemiology” phenomenon [17, 18]. The LOOK-AHEAD trial randomized 5145 overweight or obese T2D patients to an intensive lifestyle intervention that promoted weight loss (through decreased caloric intake and increased physical activity) (intervention group) or to receive diabetes support and education (control group). Patients in the intervention group lost 8.6% of their body weight in the first year vs. 0.7% in the control group. Patients in the intervention group also had greater reductions in glycated hemoglobin, and improvements in fitness and cardiovascular risk factors, without increase on morbidity and mortality [9]. Despite lower risk population in the LOOK-AHEAD trial compared with EXAMINE, these findings suggest that intentional weight loss does not increase the mortality risk; findings also supported by smaller “mechanistic” studies where weight loss was intentional, either induced by exercise training or by drugs that also improve cardiovascular outcomes, such as SGLT2 inhibitors [19, 20].

Although in EXAMINE we could not ascertain whether weight loss was intentional or unintentional, it is possible that the weight loss that drove the association with an increased risk of events represent unintentional weight losses since patients experiencing weight losses ≥ 5% had higher rates of comorbid conditions at baseline—including peripheral artery disease, atrial fibrillation, anemia, and chronic kidney disease—all of which are associated with poor outcomes either individually or in combination and across multiple populations including those with diabetes [21–24]. Together, these findings suggest that clinicians should be wary of unintentional weight loss in patients with T2D, particularly if the weight loss is in excess of 5% of body weight, where weight loss should be thoroughly investigated.

In contrast to weight loss which was strongly and independently associated with a higher risk of all cardiovascular outcomes and mortality, weight gain ≥ 5% (but not ≥ 10%) was associated only with a higher risk of HF hospitalizations, suggesting that some of the increase in body weight may reflect congestion and fluid accumulation leading to HF [25]. Other observational studies in patients with T2D found that a weight gain was associated with a lower risk of cardiovascular outcomes [26].

### Limitations

There are certain limitations to our analysis. We could not distinguish intentional from unintentional weight loss, because this information was not recorded prospectively; however, as pointed out in “Discussion” section, we believe that most of the weight loss cases were probably unintentional and potentially linked to cachexia in a population with a high burden of comorbid conditions. The findings in the study are observational and prone to confounding; therefore, causality cannot be established. Some cancer diagnoses might have passed unnoticed, and it is difficult to ascertain their contribution to the mortality seen in our study. The EXAMINE trial was published in 2013, a time where therapies that improve cardiovascular outcomes of patients with type 2 diabetes (e.g., SGLT2 inhibitors and GLP1 receptor agonists) where not available yet; therefore, our findings may not be applicable to more contemporary cohorts. Echocardiography was not performed in EXAMINE, which could have provided more insight on the underlying causes of weight gain which could represent worsening heart failure. The median follow-up of EXAMINE was relatively short (1.6 years), a longer follow-up time could have provided more information on the aggravation of chronic conditions, more frequent weight changes, and outcome events.

## Conclusions

In patients with T2D who had a recent acute coronary syndrome, a weight loss of greater than 5% of body weight was associated with a higher risk of subsequent cardiovascular events and mortality.

## Supplementary Information

**Additional file 1:** **Table S1.** Baselinecharacteristics of the patients by 10% weight change from baseline. **Table S2.** Effect of alogliptin onweight change ≥5% and ≥10%. **Table S3.**Association of body mass index with outcomes. **Figure S1.** Histogram representing the weight changes throughout thefollow-up.

## Data Availability

The data and materials may be available upon reasonable request.
