# Supplementary material for: Body weight changes in patients with type 2 diabetes and a recent acute coronary syndrome: an analysis from the EXAMINE trial
Source: Cardiovasc Diabetol. 2021 Sep 14;20:187. doi: 10.1186/s12933-021-01382-8 (PMC8442423; doi:10.1186/s12933-021-01382-8)

**Additional Material**

Additional Table S1. Baseline characteristics of the patients by 10% weight change from baseline

| **Characteristic** | **Weight loss ≥10%** | **Stable weight** | **Weight gain ≥10%** | **P-value** |
| --- | --- | --- | --- | --- |
| N. | 322 | 4461 | 597 |  |
| Age, years | 62.0 ± 10.5 | 61.1 ± 9.9 | 58.9 ± 9.5 | <0.001 |
| Age >65yr | 126 (39.1%) | 1628 (36.5%) | 153 (25.6%) | <0.001 |
| Female, n. (%) | 126 (39.1%) | 1405 (31.5%) | 198 (33.2%) | 0.015 |
| Diabetes duration, years | 6.8 (2.6, 13.9) | 7.1 (2.8, 13.7) | 7.4 (2.4, 14.0) | 0.92 |
| Glycated hemoglobin, % | 8.0 ± 1.1 | 8.0 ± 1.1 | 8.1 ± 1.1 | 0.024 |
| Insulin, n. (%) | 92 (28.6%) | 1296 (29.1%) | 217 (36.3%) | 0.001 |
| Metformin, n. (%) | 225 (69.9%) | 2970 (66.6%) | 367 (61.5%) | 0.017 |
| Sulfonylureas, n. (%) | 129 (40.1%) | 2104 (47.2%) | 270 (45.2%) | 0.038 |
| Thiazolidinediones, n. (%) | 12 (3.7%) | 103 (2.3%) | 16 (2.7%) | 0.26 |
| Weight at baseline, Kg | 86.1 ± 21.6 | 82.9 ± 19.0 | 74.7 ± 17.3 | <0.001 |
| BMI at baseline, Kg/m2 | 31.1 ± 6.4 | 29.6 ± 5.5 | 27.4 ± 5.1 | <0.001 |
| Race |  |  |  | 0.002 |
| White | 235 (73.0%) | 3275 (73.4%) | 399 (66.8%) |  |
| Asian | 68 (21.1%) | 884 (19.8%) | 137 (22.9%) |  |
| Black | 16 (5.0%) | 165 (3.7%) | 35 (5.9%) |  |
| Other | 3 (0.9%) | 137 (3.1%) | 26 (4.4%) |  |
| Current smoker, n. (%) | 43 (13.4%) | 614 (13.8%) | 77 (12.9%) | 0.84 |
| Hypertension, n. (%) | 283 (87.9%) | 3745 (83.9%) | 441 (73.9%) | <0.001 |
| HF history, n. (%) | 97 (30.1%) | 1276 (28.6%) | 160 (26.8%) | 0.53 |
| Prior stroke, n. (%) | 24 (7.5%) | 318 (7.1%) | 46 (7.7%) | 0.87 |
| PAD, n. (%) | 36 (11.2%) | 429 (9.6%) | 49 (8.2%) | 0.32 |
| Atrial Fibrillation, n. (%) | 38 (11.8%) | 302 (6.8%) | 36 (6.0%) | 0.002 |
| MI (index event), n. (%) | 248 (77.0%) | 3403 (76.5%) | 501 (84.1%) | <0.001 |
| Unstable angina (index event), n. (%) | 74 (23.0%) | 1045 (23.5%) | 95 (15.9%) |  |
| Hemoglobin, g/dL | 13.2 ± 1.6 | 13.5 ± 1.6 | 13.2 ± 1.5 | <0.001 |
| Anemia, n. (%) | 95 (29.7%) | 1091 (24.5%) | 182 (30.5%) | 0.001 |
| eGFR, ml/min/1.73m2 | 65.3 ± 20.3 | 71.0 ± 21.2 | 73.3 ± 23.3 | <0.001 |
| eGFR <60 ml/min, n. (%) | 114 (35.4%) | 1289 (28.9%) | 162 (27.1%) | 0.025 |
| Heart rate, bpm | 72.2 ± 11.9 | 71.1 ± 10.7 | 72.5 ± 10.8 | 0.006 |
| SBP, mmHg | 129.0 ± 18.6 | 129.4 ± 16.3 | 125.8 ± 18.0 | <0.001 |
| SBP >140/90 mmHg, n. (%) | 78 (24.2%) | 909 (20.4%) | 93 (15.6%) | 0.004 |
| Antiplatelets at baseline, n. (%) | 304 (94.4%) | 4346 (97.4%) | 582 (97.5%) | 0.006 |
| Beta-blockers at baseline, n. (%) | 250 (77.6%) | 3674 (82.4%) | 487 (81.6%) | 0.100 |
| Statins at baseline, n. (%) | 287 (89.1%) | 4031 (90.4%) | 548 (91.8%) | 0.38 |
| CCBs at baseline, n. (%) | 73 (22.7%) | 1025 (23.0%) | 99 (16.6%) | 0.002 |
| Diuretics at baseline, n. (%) | 144 (44.7%) | 1642 (36.8%) | 228 (38.2%) | 0.017 |
| ACEi or ARB at baseline, n. (%) | 270 (83.9%) | 3673 (82.3%) | 468 (78.4%) | 0.042 |
| Randomization to alogliptin | 159 (49.4%) | 2250 (50.4%) | 292 (48.9%) | 0.75 |

Legend: BMI, body mass index; PAD, peripheral artery disease; MI, myocardial infarction; ACS, acute coronary syndrome; eGFR, estimated glomerular filtration rate; SBP, systolic blood pressure; CCBs, calcium channel blockers; ACEi/ARBs, angiotensin converting enzyme inhibitors/angiotensin receptor blockers.

Additional Table S2. Effect of alogliptin on weight change ≥5% and ≥10%

| **Alogliptin** | **Beta (95%CI)** | **P-value** |
| --- | --- | --- |
| ≥5% weight loss | 0.15 (-0.06 to 0.36) | 0.15 |
| ≥10% weight loss | 0.01 (-0.41 to 0.41) | 0.99 |
| ≥5% weight gain | 0.05 (-0.11 to 0.21) | 0.57 |
| ≥10% weight gain | -0.03 (-0.33 to 0.26) | 0.82 |

Additional Table S3. Association of body mass index with outcomes

| **BMI category (kg/m^2^)** | **Crude HR (95%CI)** | **Adj. HR (95%CI)** | **P-value** |
| --- | --- | --- | --- |
| **All-cause mortality** | | | |
| ≤22 | 3.04 (2.17-4.29) | 2.07 (1.29-3.30) | 0.002 |
| 23-25 | 1.25 (0.90-1.74) | 1.08 (0.74-1.57) | 0.69 |
| 26-30 | Ref. | Ref. | - |
| 31-35 | 0.83 (0.61-1.13) | 0.92 (0.64-1.34) | 0.68 |
| >35 | 1.17 (0.85-1.62) | 1.36 (0.75-2.46) | 0.31 |
| **Cardiovascular mortality** | | | |
| ≤22 | 2.86 (1.90-4.30) | 1.78 (1.01-3.16) | 0.047 |
| 23-25 | 1.30 (0.89-1.90) | 1.08 (0.70-1.67) | 0.73 |
| 26-30 | Ref. | Ref. | - |
| 31-35 | 0.85 (0.59-1.21) | 0.98 (0.63-1.52) | 0.92 |
| >35 | 1.21 (0.83-1.76) | 1.55 (0.77-3.13) | 0.22 |
| **Cardiovascular mortality or heart failure hospitalization** | | | |
| ≤22 | 2.49 (1.78-3.49) | 2.30 (1.48-3.57) | <0.001 |
| 23-25 | 1.30 (0.96-1.75) | 1.32 (0.94-1.85) | 0.10 |
| 26-30 | Ref. | Ref. | - |
| 31-35 | 0.84 (0.64-1.11) | 0.73 (0.52-1.03) | 0.070 |
| >35 | 1.51 (1.15-1.98) | 0.97 (0.55-1.72) | 0.92 |
| **Primary outcome** | | | |
| ≤22 | 1.61 (1.19-2.16) | 1.80 (1.28-2.54) | 0.001 |
| 23-25 | 0.92 (0.71-1.18) | 1.03 (0.79-1.35) | 0.82 |
| 26-30 | Ref. | Ref. | - |
| 31-35 | 0.95 (0.77-1.16) | 0.75 (0.60-0.96) | 0.020 |
| >35 | 1.11 (0.88-1.39) | 0.61 (0.41-0.92) | 0.017 |

Legend: BMI, body mass index.

The primary outcome is a composite of cardiovascular death, nonfatal myocardial infarction or nonfatal stroke.

Additional Figure S1. Histogram representing the weight changes throughout the follow-up.


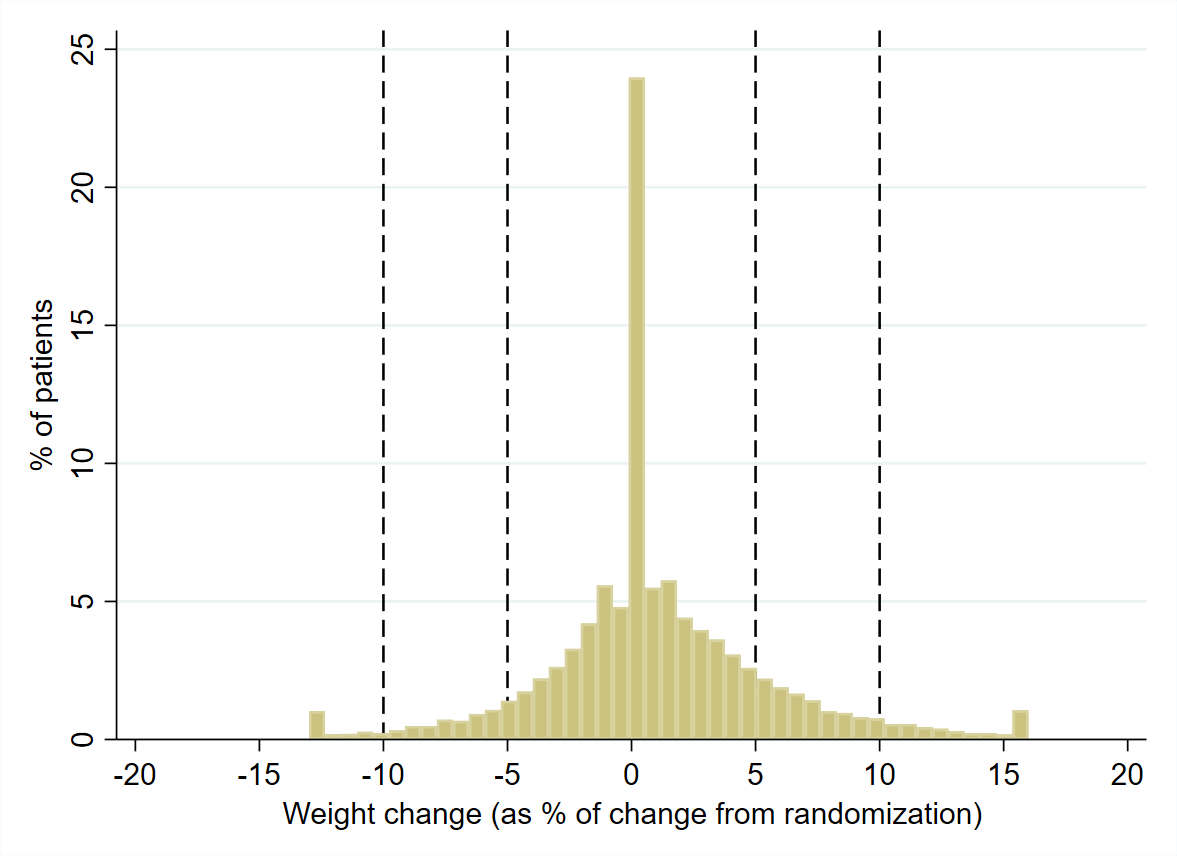

Supplement: Supplementary file 1 — Additional file 1: Table S1. Baselinecharacteristics of the patients by 10% weight change from baseline. Table S2. Effect of alogliptin onweight change ≥5% and ≥10%. Table S3.Association of body mass index with outcomes. Figure S1. Histogram representing the weight changes throughout thefollow-up. [file 12933_2021_1382_MOESM1_ESM.docx]
